# Stakeholder’s Assessment of the Awareness and Effectiveness of Smoke-free Law in Thailand

**DOI:** 10.15171/ijhpm.2018.47

**Published:** 2018-06-03

**Authors:** Nipapun Kungskulniti, Siriwan Pitayarangsarit, Stephen L. Hamann

**Affiliations:** ^1^Faculty of Public Health, Mahidol University, Bangkok, Thailand.; ^2^Center of Excellence on Environmental Health and Toxicology, Bangkok, Thailand.; ^3^Tobacco Control Research and Knowledge Management Center, Mahidol University, Bangkok, Thailand.; ^4^International Health Policy Programme, Bangkok, Thailand.

**Keywords:** WHO, FCTC, Article 8, Stakeholders, Thailand

## Abstract

**Background:** This study reports stakeholders’ ratings, and perceived gaps in World Health Organization’s (WHO) Framework Convention on Tobacco Control (FCTC) Article 8 implementation in Thailand viewed against WHO’s Guidelines for Article 8 and to inform action in preparing the 2017 Tobacco Product Control Act.

**Methods:** Stakeholder ratings of Guideline provisions of Article 8 on a three-tiered scale of implementation from understanding to effectiveness and efficiency were used to identify gaps in enforcement and compliance important to success in meeting Article 8 goals. This stakeholder assessment occurred through a stakeholder meeting of 55 stakeholders in Bangkok, Thailand in June 2016.

**Results:** The average of all assessment ratings by stakeholders on an ascending 0-3 scale had a mean score of 1.67, which means the level of implementation for Article 8 in Thailand was rated less than effective for enforcement. The assessment shows that the public understanding of smoke-free principles is also poor at a mean of 1.28, that there is incomplete effectiveness of smoke-free measures with a mean of 1.75, and only a general effectiveness that smoke-free protections are adequately covering most places with a mean of 1.98. More needs to be done to make all places compliant through enforcement efforts rated with a mean of only 1, and that more is necessary for protection from tobacco-smoke exposure in other public places and in private vehicles with mean ratings of 1.71 and 1.14.

**Conclusion:** This stakeholder approach using a three-tiered rating scale found that the implementation of Article 8 in Thailand is still lacking. With this approach, stakeholders identified critical issues needing improvement and informed changes in the then-proposed Tobacco Product Control Act which later was adopted in 2017.

## Background

The World Health Organization’s (WHO) Framework Convention on Tobacco Control (FCTC), adopted in 2003 and effective in 2005, has been a dramatic instrument of change for the tobacco control community worldwide.^1^ It has pushed tobacco use into the spotlight of health advocacy at a time when non-communicable diseases (NCDs) have emerged as the greatest threat to disease, disability, and death worldwide.^2^ The FCTC has launched a multi-factor attack on tobacco use based on established research evidence of tobacco-related disease impacts.^1^ As such, it is important to note that the parties of the Convention, known as the Conference of the Parties (COP), began their adoption of Guidelines against tobacco use with Article 8, Protection from Exposure to Tobacco Smoke.^3^

Thus, one important way to assess commitment to the FCTC is the way member states, countries who ratified the Convention, have responded to Article 8 Guidelines. The FCTC itself does not specify a timeline for member states to meet the requirements of Article 8. However, the COP-adopted guidelines do call for member nations to adopt comprehensive smoke-free policies within 5 years of FCTC adoption.^4^

Thailand has been a leader among middle-income countries in tobacco control activities in Asia. Thailand’s Non-smokers’ Health Protection Act of 1992 was Thailand’s primary legislation for the adoption of smoke-free regulations.^5^ Through that adoption and many updates of its ministerial regulations, Thailand has been recognized as a 100% smoke-free country.^5,6^ Between 2008 and 2011, Thailand implemented “Towards 100% Smoke-Free Thailand,” a program that promoted awareness and enforcement of Thai smoke-free regulation, addressing the superficial public understanding of the smoke-free law and better preparing Thai officials in more than 20 provinces to understand and implement provisions specified in the FCTC Article 8 Guidelines.^7^ Subsequently, 2014 WHO MPOWER report indicated that in 2010, Thailand had attained the highest level of worldwide achievement of Article 8, “Protection of people from tobacco smoke.”^4^

The rapid achievement of 100% smoke-free public places in Thailand has been accomplished because of several kinds of activity and commitment, including through legislation, education, and research.^8^ A 2016 Smoke-Free Index by the Southeast Asia Tobacco Control Alliance reviewed progress in 10 countries in Southeast Asia, including Thailand. It showed that Thailand’s progress is good in most areas, but that enforcement strategies, infrastructure and compliance needs improvement.^9^ As a middle-income country, Thailand is like many other countries who moved ahead on this first Article where there were guidelines for implementation. But now that guidelines on other articles have been developed, worldwide action on Article 8 has declined. Between 2010 and 2012, the number of countries implementing Article 8 increased by 15%, the most for any FCTC article. However, between 2012 and 2014, the average increase dropped to only 3%, less increase than for five other FCTC articles in the same period.^4^ Importantly, more careful attention is now being given to assessing the level of enforcement and compliance in obtaining smoke-free environments as specified in Article 8.^10^

Thailand recently proposed and passed a stronger tobacco control law, the 2017 Tobacco Products Control Act which went into effect in July 2017.^11^ In preparation for better regulatory coverage, strategies, and infrastructure for implementing smoke-free places, a stakeholder assessment for Article 8 of the FCTC in Thailand was undertaken at a one-day stakeholder meeting in 2016. Attendees were 55 government and non-government organization representatives involved in FCTC implementation and rated provisions of Article 8 implementation in Thailand at present. A three-tiered criterion of understanding, effectiveness, and efficiency was used in giving the ratings. This study reports stakeholders’ ratings, and perceived gaps in WHO-FCTC Article 8 implementation in Thailand viewed in relation to Article 8 Guidelines and to inform action in preparing the 2017 Tobacco Product Control Act.

## Methods

The Tobacco Control Research and Knowledge Management Center (TRC), a tobacco control research unit funded by the Thai Health Promotion Foundation initiated a stakeholder assessment of major Articles of the WHO FCTC including Article 8 in 2016. To provide a framework for rating implementation, a three-tiered scale with scoring between 0 and 3 to establish the level of implementation of provisions of Article 8 was developed by tobacco control experts who had worked with TRC on FCTC research. The stakeholder approach was chosen since it is an accepted method of confirming community concerns needing further investigation through research.^12^

A stakeholder meeting was held with representatives from six Ministries, National Police Office, Bangkok Metropolitan Administration and non-government organizations including experts and experienced officials with a familiarity of the implementation of the smoke-free law and its enforcement in Thailand. In the meeting, ratings were given based on stakeholders’ views of the situation in Thailand in 2016 and then the ratings were averaged for the final ratings of Article 8 provisions as in Figure.

**Figure:**
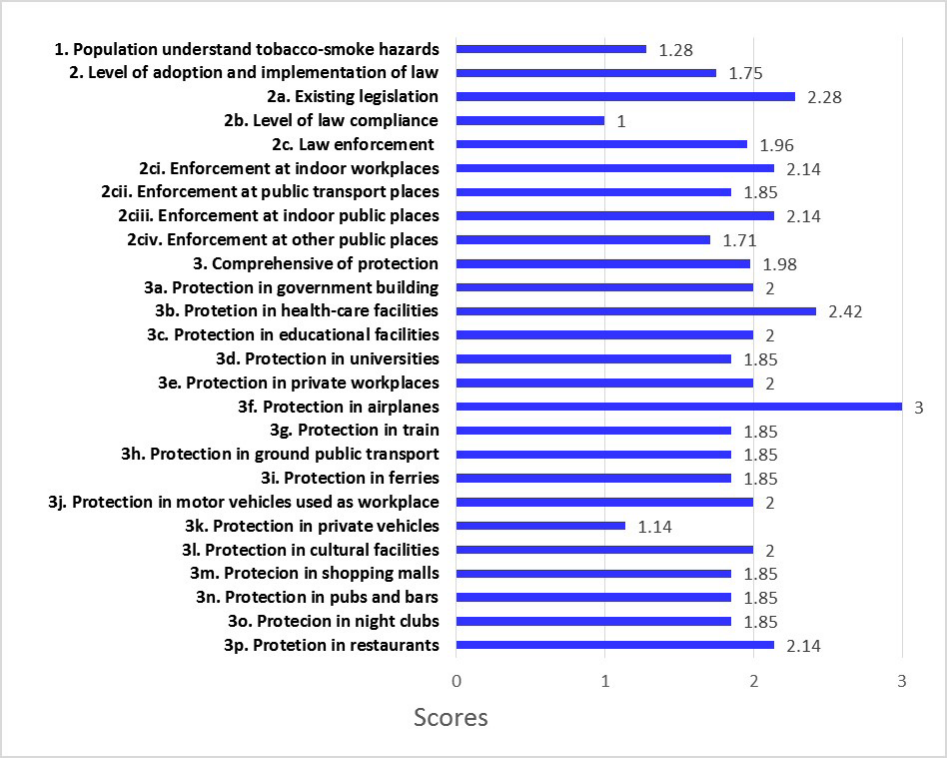
Article 8 Items and Assessment Ratings by Stakeholders for Implementation of Article 8 in Thailand. Note: There are three main Article areas shown: education, legislation and enforcement with coverage of public spaces.

In the ratings which followed the meeting, 0 meant “not understood or effective.” A rating of 1 meant “understood but not effective,” whereas a rating of 2 meant “effective but not efficient.” Finally, a rating of 3 signified “highly effective and efficient” implementation. The 26 rated items for Article 8 of the WHO FCTC for the protection from exposure to tobacco smoke were assessed as to current practice in 2016 in Thailand and were from the Guidelines for implementation of the WHO Framework Convention on Tobacco Control, 2013.^3^

## Results

Figure provides the results of this implementation assessment. The ratings of items in Figure reflect stakeholder views of public understanding of smoke-free principles not understood (level 1), law effectiveness (compliance) (level 2) and law effectiveness and efficiency (level 3). As shown in Figure, the public, as assessed by stakeholders, has a poor understanding of tobacco smoke harms (1.28), there is incomplete effectiveness of smoke-free measures (mean of 1.75), and there is general satisfaction that smoke-free protections are effectively comprehensive, covering most places (mean of 1.98). The mean score of 1.75 for item 2 is the average of three items about the adoption and implementation of measures/laws to protect from exposure to tobacco smoke including law compliance and enforcement. The level of compliance with smoke-free laws was scored lowest at 1. For item 3 on the comprehensive protection from exposure to tobacco smoke in all places and areas, a mean score of 1.98 is obtained from the level of protection in 16 areas (3a-3p). Satisfaction of effectiveness was present with the level of protection in government buildings, healthcare and educational facilities, private workplaces, on airplanes, in motor vehicles used for work, in cultural facilities and restaurants, all rated 2.0 or above. However, half of the ratings of places did not reach level 2.0 of effective compliance.

## Discussion and Recommendation

The mean of all Article 8 assessment ratings from Figure is 1.67, which means the level of implementation rated for effectiveness in Thailand is still low. More needs to be done to make all places comply with enforcement efforts. Even though some past studies^13-15^ reported that the Thai public had shown a high level of understanding that secondhand smoke is dangerous, stakeholders perceive this understanding to be quite superficial.

Also, the score of 1.71 (2civ) shows inadequate implementation for protection from exposure to tobacco smoke in other public places. The need for attention to these other places where people are crowded together is indicated by this rating.

Significantly, a score of 1.14 (3k) was given for protection in private vehicles. This score is the lowest, indicating lack of protection as compared to other specific places listed in item 3 since Thailand has not yet banned smoking in private cars. Many countries already have smoking bans in private vehicles to protect passengers from secondhand smoke and to improve road safety, especially to protect children from smoke that can cause them both short- and long-term health effects.^16^

Another concern of importance to stakeholders was smoking at entrances to public buildings where smoking is prohibited inside. The recommendations reported below reflect stakeholder concerns and have informed consideration of the provisions of the proposed Tobacco Product Control Act which subsequently was adopted in 2017.

The rating results confirm various concerns and gaps observed by stakeholders and requiring a public response. Some responses were expressed as recommendations requiring long-term actions while enforcement actions were seen as more immediately possible.

While the stakeholder approach is useful in capturing ‘bottom-up’ realities that may not be clear from the national level and is useful in checking the validity of research priorities, it still has several limitations. Stakeholders are limited in number and may not be representative enough for generalizability. There may also be a reluctance by stakeholders to openly express their opinions, especially criticisms, of present implementation, coordination, resources and achievements in a meeting where multiple organizations are present. Because the stakeholder meeting was held before the adoption of legislative improvements through a new law, finally passed in 2017, it was timely that the meeting could be framed as a means for improvements to implementation.^17^ The meeting results characterized here may not have reflected a consensus of all stakeholder views in Thailand, but did seem to identify some main themes consistent with other assessments that spoke to specific implementation gaps such as lack of human resources.^18^

Recommendations of principle actions flowing from this stakeholder  assessment  are:

- Provide education on secondhand and third-hand smoke hazards via various media, social networks and health professionals to target groups, as indicated by the rating on Item 1 of Figure.
- Expand the legal authority and enforcement resources necessary to yield efficient protection of the public from exposure to tobacco smoke in all public and other locations.
- Initiate regular field inspections for detection of smoking violations of the smoke-free law.
- Lawbreakers, both individuals, and places, should be assessed fines and/or sentences of incarceration for violation of smoke-free laws.
- Adopt legal measures to prohibit smoking in private vehicles when children are present.
- Adopt legal measures to prohibit smoking within a distance of 10 meters from entry ways of buildings and on the grounds of places where smoking is prohibited.^19,20^

## Conclusion

Implementation assessment of Article 8 of the FCTC reflects both the government’s overall seriousness about the dangers of exposure to tobacco smoke and the general public’s willingness to accept an environment where freedom from such exposure is adopted as the norm in society. While specific provisions of an international treaty for health may be a distant concern of persons with local concerns, stakeholders who feel the needs and aspirations of community members often can provide insight to behaviours of most relevance to them. Specifically, local processes of awareness and practical endorsement were most in evidence. The contribution of stakeholders through this assessment has shown how the implementation of multiple levels can inform research and policy to help Thailand in its continued quest for complete Article 8 compliance.

## Acknowledgements

This study is a part of the work by the TRC working group for FCTC assessment in Thailand. The authors thank Duangkamon Sritabutra and Ratikorn Pembrige of TRC who coordinated the workshop and collection of data.

## Ethical issues

The ethical issue is not applicable since our paper was based on the policy report of Stakeholders’ meetings for the assessment of the awareness and effectiveness of smoke-free law in Thailand to the Tobacco Control Research and Knowledge Management Center.

## Competing interests

Authors declare that they have no competing interests.

## Authors’ contributions

SP designed the study, coordinated the workshop for data collection. NK developed the assessment items and description, facilitated the stakeholder meeting, and drafted the manuscript. SH assisted with the writing and revision of the manuscript. All authors finalized the manuscript.

## Authors’ affiliations

^1^Faculty of Public Health, Mahidol University, Bangkok, Thailand. ^2^Center of Excellence on Environmental Health and Toxicology, Bangkok, Thailand. ^3^Tobacco Control Research and Knowledge Management Center, Mahidol University, Bangkok, Thailand. ^4^International Health Policy Programme, Bangkok, Thailand.

### Key messages

**Implications for policy makers**

A policy blueprint for policy-makers based on this study includes:

- Examining Framework Convention on Tobacco Control (FCTC) compliance through the stakeholder approach brings the realities of local implementation forward for policy-makers and researchers.
- Limitations to policy effectiveness and efficiency are often most clearly identified from the bottom up regarding coordination and available resources.

**Implications for the public**

Public policy meets the needs of the public when it addresses several understandings of implementation. It commonly must be rational (evidence-based), balanced (addressing competing interests) and highly practical (actionable). Looking at the implementation in this way gives the public assurance that policy is being devised with the total context of societal needs in mind.

## References

[1] Nikogosian H, Kickbusch I (2016). The Legal strength of international health instruments - What it brings to global health governance?. Int J Health Policy Manag.

[2] Puska P (2017). WHO FCTC as a pioneering and learning instrument; Comment on “The legal strength of international health instruments - What it brings to global health governance?”. Int J Health Policy Manag.

[3] World Health Organization. Guidelines for implementation Article 5.3, Article 8, Article 9 and 10, Article 11, Article 12, Article 13, Article 14. WHO Framework Convention on Tobacco Control; 2013.

[4] World Health Organization. 2014 Global Progress Report on Implementation of the WHO Framework Convention on Tobacco Control. WHO Framework Convention on Tobacco Control; 2014.

[5] Ministry of Public Health. Tobacco Product Control Act B.E.2535 (1992) and Non-Smokers’ Health Protection Act B.E.2535 (1992). Printed as a supplement for Conference of Parties II (COP II). Group of Tobacco and Alcohol Consumption Control, Department of Disease Control, Nonthaburi, Thailand: Ministry of Public Health; 2007.

[6] World Health Organization. WHO report on the global tobacco epidemic 2017: Monitoring tobacco use and prevention policies. Geneva: Switzerland: 2017.

[7] The International Union against Tuberculosis and Lung Disease. Final End of Project Completion Report: Towards 100% Smoke-Free Environment, Thailand. http://btc.ddc.moph.go.th/th/upload/datacenter/data7.pdf. Accessed April 10, 2017. Published 2012.

[8] Hamann SL, Mock J, Hense S, Charoenca N, Kungskulniti N (2012). Building tobacco control research in Thailand: meeting the need for innovative change in Asia. Health Res Policy Syst.

[9] Southeast Asia Tobacco Control Alliance. Smoke-free Index: Implementation of Article 8 of the WHO Framework Convention on Tobacco Control. Bangkok, Thailand: SEATCA; 2016.

[10] Cairney P, Mamudu HM. The WHO Framework Convention for Tobacco Control (FCTC): What would have to change to ensure effective policy implementation? Published September 18, 2013.

[11] Royal Thai Government. Tobacco Products Control Act, B.E. 2560. Book 134, Section 39. Bangkok: The Government Gazette; 2017.

[12] Schur CL, Berk ML, Silver LE, Yegian JM, O’Grady MJ (2009). Connecting the ivory tower to main street: setting research priorities for real-world impact. Health Aff (Millwood).

[13] World Health Organization. Global Adult Tobacco Survey (GATS): Thailand Country Report. Regional Office for South-East Asia: World Health Organization; 2009.

[14] Yong HH, Foong K, Borland R (2010). Support for and reported compliance among smokers with smoke-free policies in air-conditioned hospitality venues in Malaysia and Thailand: findings from the International Tobacco Control Southeast Asia Survey. Asia Pac J Public Health.

[15] Lapvongwatana P, Kungskulniti N, Charoenca N, Avila-Tang E, Wipfli H, Hamann SL (2016). A Cross-sectional Study of Secondhand Smoke Exposure among Non-smoking Women and Children in Thai Households. Environ Nat Resour J.

[16] Wikipedia. Smoking bans in private vehicles. https://en.wikipedia.org/wiki/Smoking_bans_in_private_vehicles. Accessed January 26, 2018. Last modified January 21, 2018.

[17] O’Haire C, McPheeters M, Nakamoto E, et al. Engaging stakeholders to identify and prioritize future research needs. Rockville, Maryland: Agency for Healthcare Research and Quality (US); 2011. 21977526

[18] World Health Organization. WHO report on the global tobacco epidemic 2009: Implementing smoke-free environments. Geneva, Switzerland: WHO; 2009.

[19] Kaufman P, Zhang B, Bondy SJ, Klepeis N, Ferrence R (2011). Not just ‘a few wisps’: real-time measurement of tobacco smoke at entrances to office buildings. Tob Control.

[20] Hwang J, Lee K (2014). Determination of outdoor tobacco smoke exposure by distance from a smoking source. Nicotine Tob Res.

